# Walk-weighted subsequence kernels for protein-protein interaction extraction

**DOI:** 10.1186/1471-2105-11-107

**Published:** 2010-02-25

**Authors:** Seonho Kim, Juntae Yoon, Jihoon Yang, Seog Park

**Affiliations:** 1 Department of Computer Science, Sogang University, Seoul, Korea; 2 Daumsoft Inc, Se-Ah Venture Tower, Seoul, Korea

## Abstract

**Background:**

The construction of interaction networks between proteins is central to understanding the underlying biological processes. However, since many useful relations are excluded in databases and remain hidden in raw text, a study on automatic interaction extraction from text is important in bioinformatics field.

**Results:**

Here, we suggest two kinds of kernel methods for genic interaction extraction, considering the structural aspects of sentences. First, we improve our prior dependency kernel by modifying the kernel function so that it can involve various substructures in terms of (1) e-walks, (2) partial match, (3) non-contiguous paths, and (4) different significance of substructures. Second, we propose the walk-weighted subsequence kernel to parameterize non-contiguous syntactic structures as well as semantic roles and lexical features, which makes learning structural aspects from a small amount of training data effective. Furthermore, we distinguish the significances of parameters such as syntactic locality, semantic roles, and lexical features by varying their weights.

**Conclusions:**

We addressed the genic interaction problem with various dependency kernels and suggested various structural kernel scenarios based on the directed shortest dependency path connecting two entities. Consequently, we obtained promising results over genic interaction data sets with the walk-weighted subsequence kernel. The results are compared using automatically parsed third party protein-protein interaction (PPI) data as well as perfectly syntactic labeled PPI data.

## Background

### Introduction

In recent years, biomedical research has been accelerated by technological advances in biomedical science and a surge of genomic data from the Human Genome Project and a huge amount of new information has been coming from the related research. In order to manage the proliferation of biomedical data, databases such as SWISS-PROT [1], BIND [2], MINT [3], and UniProt [4] have been developed. However, since a majority of databases still rely on human curators, substantial manual efforts are required to cope with enormous collections on biomedical research and publications. Thus, the development of high-quality information extraction tools that allow scientists and curators to quickly access new discoveries is an important issue in bioinformatics.

**Figure 1:**
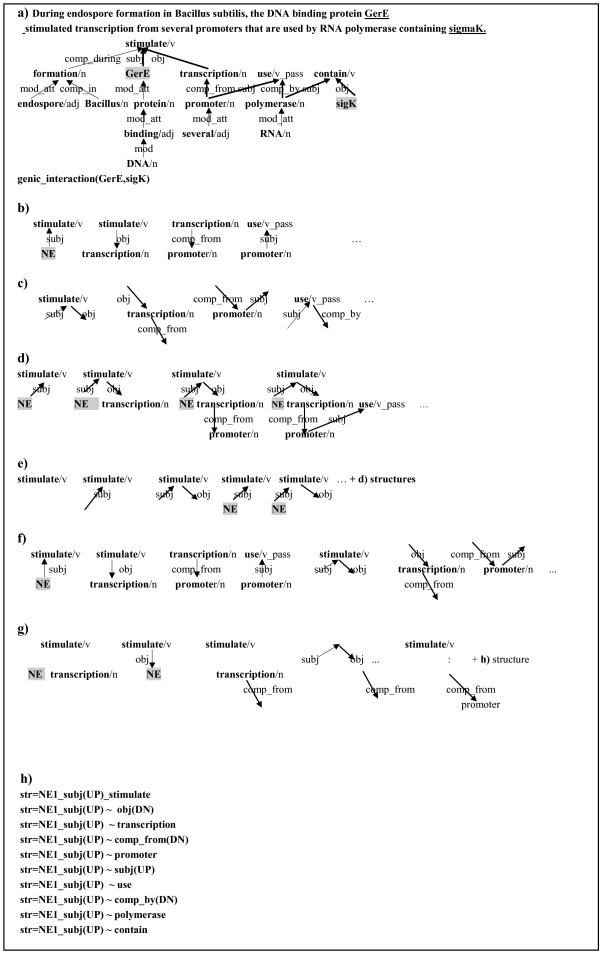
**Substructures for kernels**. A complex sentence with long range relations and substructures which are considered in each kernel are shown. a) a sentence with long range relations (bold →: shortest path), b) examples of v-walk, c) examples of e-walks, d) substructures considered in previous dependency kernel, e) substructures considered in extended dependency kernel, f) substructures for spectrum kernel, g) substructures for fixed-length subsequence kernel.

Biomedical information extraction usually involves the recognition of biomedical entities and pre-defined types of facts, such as relations/interactions between the entities, through the analysis of raw textual data. While there has been substantial progress in biomedical named entity recognition, relation/interaction extraction is still challenging since biomedical texts often contain complex sentences with long-range relations, as shown in Figure 1a.

One major approach to this issue is to adopt specific types of matching rules or patterns as the core relation discovery operation. The patterns are mainly represented in the form of sequences of words, parts-of-speech (POS), or syntactic constituents [5,6]. Such pattern-based relation extraction can provide an intuitively easy methodology with high precision, but pattern forms are too rigid to capture semantic/syntactic paraphrases or long-range relations, which lead to low recall rates. Thus, some works suggested more generalized pattern learning methods to align relevant sentences [7-9].

As an alternative, various kernel methods have been employed to this relation extraction problem. Such methods have, in particular, provided appealing solutions for learning rich structural data such as syntactic parse trees and dependency structures, which cannot be easily expressed via the flat features. Kernels are normally designed to capture structural similarities between instances based on the common substructures they share. Mostly, the similarities between structures can be efficiently computed in a recursive manner without explicitly enumerating with feature vectors, which enables us to avoid complex feature construction and selection processes [9-14].

### Motivation

This work expands on our previous kernel approaches [13]. Previously, we had addressed problems of genic and PPI extraction between biomedical entities with four kernel methods: predicate kernel and walk kernel (feature-based), dependency kernel (structure-based kernel), and hybrid kernel (composite kernel of structure-based and feature-based kernels). Each kernel captured structural information in a different way to find the relationships between genes/proteins in a sentence. The kernels are based on the shortest path connecting two entities on the syntactic parse trees (dependency graph). We explored the interaction learning problem in the following aspects: (1) efficient data representation, (2) what semantic/syntactic features or substructures on the shortest path linking interactive two entities are useful for relation learning, and (3) how those structures can be incorporated into kernels. The results revealed that the walk kernel, one of the feature-based kernels, showed a very competitive performance on Learning Language in Logic (LLL) data [15], with an F-score of 77.5.

In the feature-based kernels, the syntactic substructures of relation instances were mapped to flat features. For example, the walk kernel learned interactions through *v*-walk and *e*-walk features on the shortest dependency paths. In that kernel, a *v*-walk feature consisted of (*word*_*1*_, *relation, word*_*2*_) and (*POS*_*1*_, *relation, POS*_*2*_), and an *e*-walk feature was composed of (*relation*_*1*_, *word, relation*_*2*_) and (*relation*_*1*_, *POS, relation*_*2*_) where a word/POS is a node and a syntactic dependency relation between two nodes is an edge on the dependency graph, as shown in Figure 1b and Figure 1c. The dependency relation between a head and its dependent was roughly represented with seven main functions of relations: appos (apposition), comp_prep (prepositional complement), mod (modifier), mod_att (attributive modifier), neg (negation), obj (object) and subj (subject).

On the contrary, the structure-based kernel was represented by structural similarities between relation instances (See Equation (3), (4)), instead of explicit feature enumerations. However, contrary to our expectation, the kernel based on structural isomorphism between the shortest path graphs showed lower performances than the feature-based kernel.

This study starts from the drawbacks of our previous dependency kernel. It showed difficulties in handling the following aspects: (1) *e-walks*, (2) partial match, and (3) non-contiguous paths, and (4) different significance of substructures. First, in the kernel, the structural similarity between two relation instances was recursively captured by comparisons of *v-walks *(node-edge-node) on their sub-graphs. In other words, *v-walks *(Figure 1b) were compared, but *e-walks *(Figure 1c), one of the features in the walk kernel, were not. However, according to our experiments, the *e-walk *feature practically plays a more important role in determining the relation than *v-walk*. Second, fragments such as "*subj (UP) stimulate*" or "*subj (UP) stimulate obj(DN)*" shown in Figure 1e were excluded in the substructure comparisons. Thus, two subgraphs matched only when two root nodes and their direct child nodes were the same and the dependency relationships between them were the same. Such a match is referred to as a *complete path match*. It consists of a connected sub-graph with at least two words. However, the cases where a series of node-edge-nodes between two graphs are identical can be sparse. Thus, we additionally consider fragment structures in the learning framework, which are incomplete graphs like in Figure 1e. This is referred to as *partial path match*. Third, the kernel considered internally contiguous dependency substructures on the shortest path. However, non-contiguous substructures can be important in genic interaction. For example, a non-contiguous relation between two entities such as "*simulate~comp_from(DN) promoter*" in Figure 1g may have an effect on genic interaction. This substructure is called *non-contiguous path*. Finally, the kernel counted all common subgraphs equally regardless of their importance, even though some subgraphs have more useful properties for learning than others. The kernel made no distinction between the significances of structures. We tackle the 4 issues with the new kernels.

### Research goal and differences from other recent similar work

The first three substructures mentioned above can be covered by general graph kernels [14,16]. However, none of related studies treated the fourth issue from a syntactic structure perspective. The main idea is that each dependency structure has a potentially different property and significance for relation learning according to its type. Thus, we properly classify the substructures that the directed shortest path encloses and distinctively incorporated them into kernels according to the types of dependency substructures.

This point differs from other recent kernel approaches that address the PPI extraction [9,11,14,16,17]. Moreover, our system can handle directed interactions that the roles of entities are separated as agent and target, while many PPI systems assume interactions to be undirected [9,11,14,17-19].

Here, we first evaluate the effectiveness of different dependency subpaths and revise the dependency kernel so as to overcome the problems of complete subgraphs and equal counting for all subgraphs. In order to treat *non-contiguous path *substructures, we next introduce string kernels that compare two instances (paths) in terms of substrings they contain. Finally, we propose the walk-weighted subsequence kernel which assigns different weights according to the types of common substrings between two shortest path strings. That is, lexical subgraphs and morpho-syntactic subgraphs, *e-walk *and *v-walk*, and contiguous and non-contiguous dependencies are all differently handled by this kernel. In the experiments, we evaluated our kernels on the 5 PPI corpora by [18]. In addition, we compared the performances on human-annotated data and automatically parsed data.

### Related works

In general, a deeper linguistic representation is known to support information extraction well if its accuracy is guaranteed. Thus, many researches related to relation extraction have used shallow or full syntactic analysis. In particular, words between two entities are considered to carry important information regarding relationships between the entities. Furthermore, structural information of a sentence affects the relation learning. However, the use of a whole sentential structure can generate noise in learning since all constituents in a sentence actually do not concern an interaction between two entities. Thus, we need to restrict the structure for interaction learning to directly or indirectly relevant ones to two entities. One of the ways is to use the shortest path between two entities on a syntactic graph.

As an alternative to the shortest path approach, [14] suggested the all-dependency-paths kernel to identify protein/gene interactions. In order to extract correct interactions, they represented a parse tree of a sentence with a dependency graph and considered dependencies outside the shortest path connecting two entities as well as dependencies on the shortest path. They assigned a weight of 0.9 to the edges (dependencies) on the shortest path and a weight of 0.3 to other edges. Consequently, the weighting scheme helps emphasize the dependencies on the shortest path without excluding dependencies other than the shortest path. Thus, potentially relevant words outside of the shortest path can be included in the kernel.

However, [12] reported that subtrees enclosed by the shortest path between two entities still describe their relation better than other subtrees, even though the representation can miss important words outside the shortest path in some cases, as pointed by [14].

As a feature-based approach, [11] used various syntactic path features, which are encoded with SVM. They used the predicate argument structures obtained by a head-driven phrase structure grammar (HPSG) parser and a dependency parser, and word context features related to words before, between, and after two interacting NEs.

On the other hand, some works considered only shallow linguistic information concerning word context features without using structural information by parsing. [9] expressed a relation between two entities by using only words that appear in fore-between, between, and between-after the entities. They utilized neighboring words and their word class sequences to discover the presence of a relation between entities. [17] extended [9]'s work.

[16] proposed the composite kernel, which combines previously suggested kernels: the all-paths-dependency kernel of [14], the bag-of-words kernel of [11], and the subset tree kernel of [20]. They used multiple parser inputs as well as multiple kernels. The system is the current state-of-the-art PPI extraction system on various PPI corpora. They also boosted system performance by adopting the corpus weighting concept (SVM-CW) [21].

Recently, the BioNLP 2009 shared task considered more detailed behaviours of bio-molecules [22] as compared to previous PPI researches. The main task required the recognition of bio-molecular events, event types, and primary argument concerning the given proteins. The 8 event categories such as gene expression, transcription, protein catablolism, phoshorylation, localization, binding, and regulation were considered and the best result in the task was 51.59% (F-score).

### Data representation

**Figure 2:**
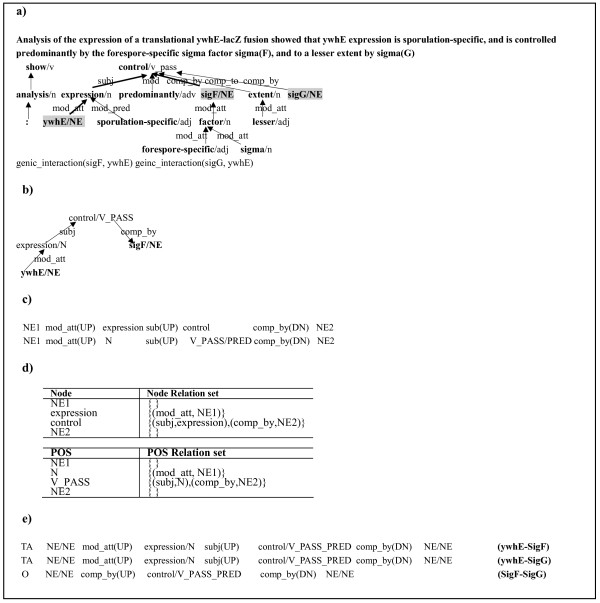
**Data Representations**. The shortest path representation between NE pairs and the shortest path string are visualized. a) dependency graph for the given sentence, b) shortest dependency path between ywhE and sigF, c) lexicalized dependency path string (up) & syntactic dependency path string (down), d) Word dependency list (up) & POS dependency list (down), e) shortest path string instances of the sentence a).

In this work, we represent a parsed sentence as a dependency graph in which the nodes denote words/POS and edges denote types of dependency relations between the nodes. We constrict an essential structure for modeling of a relationship between two entities to the directed shortest dependency path that connects them on the sentential graphs. However, to cover some limitations of the shortest path representation as pointed in [12] and [14], single paths that consist of two NEs and a single direct syntactic dependency of a coordinating conjunction between them are extended. It is sometimes insufficient to determine the relation only with the shortest path because of cases such as "*NE*_*1 *_*conj_and NE*_*2*_" when two NEs are joined by a conjunction. In fact, most cases have no interactions except some cases that co-occur with binding, association, recombinant, or interaction contexts. Thus, the single shortest paths are extended to contain predicated-head or clue context words in the path such as "*interaction between ~ and*~", "*association between ~ and *~", and "*recombinant ~ and *~" by finding the immediate head of NE and the head of its head. In addition, we add the negation mark before a predicate on the path when a negation expression occurs around the predicate. Besides, predicate and direction information to each edge of a graph are added, which can help identify directed relations, as shown in Figure 2. This representation is different from those in recently published other works that encode relations with the shortest path descriptions between entities [9,12]. It can be efficient and informative for learning.

### Directed shortest dependency path

In order to represent an interaction instance with the shortest traversal path between two entities on the parse graph, we used Dijkstra's algorithm [23]. We first transformed the dependency graph to an undirected graph which allows the edges to be traversed in any direction because every syntactic relation is toward the syntactic head. However, to preserve the original directions of the relations on the graph, we assign a dependent-head edge with "UP" and conversely, "DN" for a head-to-dependent edge. Furthermore, the shortest path string is defined as a sequence of words or parts-of-speech (POS) connected by directed dependency relations, as shown in Figure 2b. The presence of the "PRED" label for a word on the path indicates that the directions of the left or right edges connected to the word are changed. This often occurs in predicative words. Since a key component of semantic relation determination is to identify the semantic arguments filling the roles of predicates, such predicate markers can be an informative feature on predicate argument structures for a given sentence. Figure 2b visualizes the shortest dependency path linking "*ywhE*" and "*sigF*". It is reused as its path string form (Figure 2c) for string kernels and as a dependency list form (Figure 2d) for the dependency kernel. The lexicalized dependency path string consists of words and their dependency relations. We also consider the syntactic dependency path string, which consists of POS and their dependency relations, incorporating direction and predicate information. Likewise, a POS dependency list contains pairs of POS and the syntactic relations between the POS and their direct child nodes' POS. For each node on a graph, the word dependency list contains pairs of nodes and the syntactic relations between the nodes and their direct child nodes. The labels of all NE pair instances are represented in word order with "TA" (target-agent), "AT" (agent-target) and "O" (no interaction). Figure 2e shows some instances derived from the sentence Figure 2a.

### Kernel methods

For the relation learning, we basically adopt a kernel approach. Kernel means a similarity function that maps a pair of instances to their similarity score. That is, the kernel *K *over an object (feature) space *X *can be represented with a function *K*: *X *× *X *→ [0, ∞]. Thus, objects of kernel methods are expressed by a similarity matrix. In general, a kernel can be easily computed using inner products between objects without explicit feature handling. Thus, it can be operated well on a rich structured representation which has a high dimensional feature space such as graph or tree.

In this work, a genic relation pair for kernel functions is represented by the shortest dependency path between two NEs on the syntactic graph. Thus, the proposed kernels compute structural similarities in various ways according to the substructures that two dependency paths contain. Each kernel defines meaningful substructures differently.

## Results and discussion

### Train and test data

We performed experiments on extracting gene and protein interactions from two different data sets, automatically parsed and perfectly parsed data sets. First of all, we used the LLL 05 shared task data for individual evaluation of the kernels that we proposed in this work. The dataset provides dependency syntactic information and directed interactions annotated with agent and target roles. The LLL basically used the Link Grammar Parser [24] for syntactic analysis and the parsed results were manually corrected. Thus, it is a clean data set. The dependency analysis produced by the parser was simplified to 27 grammatical relations. The typed dependencies are shown in Figure 1. The task provides a separate test set and external evaluations through the web server http://genome.jouy.inra.fr/texte/LLLchallenge/scoringService.php. The task information is available at http://genome.jouy.inra.fr/texte/LLLchallenge/ and further details about the data set are described in [15].

**Figure 3:**
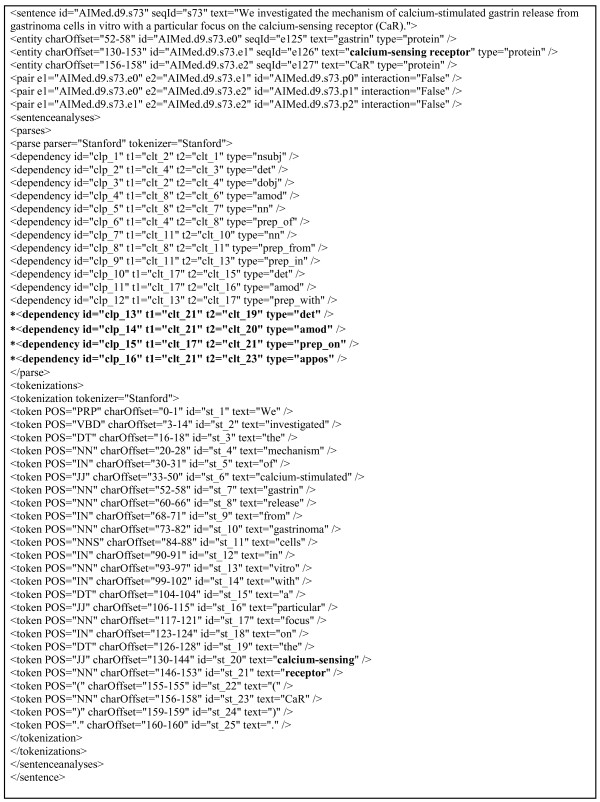
**Examples of Typed Dependencies**. The dependency information considered in the converted corpora by [18] is shown. The entity, "calcium-sensing receptor" forms separate tokens. Thus the relations marked with * should be reorganized.

The proposed kernels are also evaluated using the 5 PPI corpora which put together by [18] for comparative evaluations on diverse corpora. Many recent studies that addressed PPI task used the corpora as default benchmarks. The corpora consist of AIMed, BioInfer, IEPA, HRPD50, and LLL 05. For syntactic dependency information, we used the converted version by [18] which contains fully automated dependency parsing results, as shown in Figure 3. The corpora were parsed with Charniak and Lease parser [25] and the parsed results were transformed into the collapsed Stanford dependency scheme. As shown in Figure 3, they contain information regarding entities such as proteins/genes/RNA/chemicals, interaction pairs between entities, tokens, and syntactic dependencies between tokens in a unified format, which enable all 5 corpora to be easily integrated into a system. The typed dependencies were represented with 55 grammatical relations. Details about the corpora and their characteristics can be found in [18]. The converted corpora are available at http://mars.cs.utu.fi/PPICorpora/.

In case of the converted corpora, we confine interaction extraction to the NE pairs that [18] considered. The example size of each corpus is shown in Table 1. Meanwhile, in case of original LLL data, 464 NE pairs on the training set are used to train the kernels and 330 NE pairs on the test set are classified. We first constructed an NE dictionary and identified all interaction pairs of NEs occurring in the dictionary. Among them, the pairs, whose interactions were not stated as genic interactions, were used as negative examples because LLL provides only interactive pairs.

**Table 1:** Example size of the 5 converted PPI corpora by [18]

Corpus	Positive Examples	negative	All examples
**AIMed**	1,000	4,834	5,834
**BioInfer**	2,479	7,174	9,653
**HPRD50**	163	270	433
**IEPA**	335	482	817
**LLL**	164	166	330

The sizes of training corpora are shown.

Both the perfectly parsed and third party auto-parsed data sets are analyzed into typed dependency forms. However, two syntactic analyses for relative clauses are remarkably different from each other. In addition, the converted corpora have much more dependency types than LLL and some typed dependencies on the corpora are redundant. For example, there is no clear-cut distinction between the pronominal relation types, "*amod*" (adjectival modifier), "*nn*" (nominal modifier) and "*dep*"(dependent).

To clarify perfectly parsed LLL dataset and auto-parsed LLL dataset, we will refer to the former as "LLL" and the latter by [18] as "converted LLL". "Converted corpora" is short for the dependency-parsed 5 corpora by [18].

### Preprocessing for the third party automated inputs

Using the converted corpora, it is quite difficult to directly retrieve syntactic dependencies for the following reasons: (1) multiple syntactic relations and (2) self cycle relations between two nodes exist, and (3) NE tag information is not reflected in the parsed results, as shown in Figure 3. Self cycle here means that an edge (x, y) and its inverted edge (y, x) coexist between two nodes, x and y.

**Figure 4:**
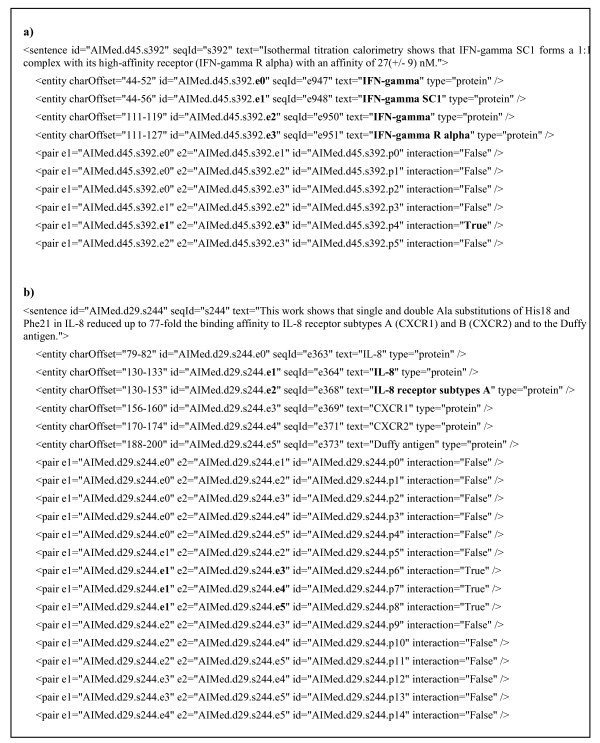
**Embedded Entities**. Some interaction examples are given regarding embedded entities. a) the longest entity is the interactive entity, b) the embedded entity is involved in the interaction.

In case of (3), each word of an NE corresponds to an individual terminal node and constitutes syntactic relations with other words. In fact, this is a main drawback of the converted data, which causes erroneous results. It requires an additional pre-processing to eliminate or join unnecessary syntactic relations connected to NE words. That is, NE words are grouped again from the converted results and dependency relations relating NE words are readjusted. In this process, multiple syntactic relations can come out again. In addition, some named entities are embedded in other named entities. Embedded (nested) named entities refer to the cases in which one entity contains another. In such cases, two entities share the same dependency structure but the interaction results should be different because one of them participates in an actual interaction. This presents one of the difficulties in the aspect of learning. Figure 4 shows some interaction examples containing embedded entities. In Figure 4a, the longest entity is the interactive entity. On the contrary, in Figure 4b, the embedded (shortest) entity is involved in the interaction. Thus, embedded NEs disturb a normal learning process by making it difficult to detect real interactive NEs. We included all embedded entities in the training and test data for comparisons with other systems.

To address the above problems, we performed a rough disambiguation process. Table 2 summarizes the numbers of multiple relations, self cycle relations, operations needed for grouping NE words, and sentences including nested NEs with respect to 5 corpora. For example, the converted AIMed includes 104 multiple relations and 309 self-cycle dependencies to be removed. Also, 114 out of 1,954 sentences contain embedded entities. Currently, extra pre-processing such as tag fixing and sentence splitting used in other works [14,16] was not performed in this study.

**Table 2:** Pre-processing

	LLL	AIMed	BioInfer	IEPA	HPRD50
**a)**	77	1,954	1,100	486	145
**b)**	1,707	36,388	25,337	11,571	2,796
**c)**	14	172	249	67	11
**d)**	20	342	230	49	16
**e)**	70	1,859	2,243	487	121
**f)**	0	115	44	0	0

The numbers of multiple relations, self cycle relations, operations (eliminate/join relations) for grouping NE words, and sentences including nested NEs, which need a pre-processing, are summarized with respect to 5 corpora.

a) total number of sentences, b) total number of syntactic relations, c) number of multiple syntactic relations, d) number of self-cycle syntactic relations, e) number of operations for grouping NE words, f) number of sentences including nested NEs.

### Learning and evaluation method

Each kernel is combined with SVM. The LIBSVM 2.84 package [26], which allows multi-class classifications and user-defined kernels, is used for SVM learning. In this study, we define our own kernels and set the C value as 1,000 for the best F-value. In addition, each kernel is normalized corresponding to the kernel space as follows [27]:(1)

Extraction performances are evaluated by the F-score. As mentioned before, we can check the performance over the LLL test data through an external evaluation. On the contrary, separate test sets and external evaluation schemes are not provided to the other 4 corpora. Thus, the proposed kernel is evaluated by the 10-fold document-level cross-validation used in many recent works [9,11,14,17]. We adopt the same data splitting and evaluation strategy as the study. Also, if the same protein name occurs multiple times in a sentence, the interactions are identified over each occurrence. However, self interactions that a single protein interacts with itself are regarded as negative, whereas in many other works, self interactions are not considered as candidates and removed prior to evaluation.

### Experimental Results

In this paper, we proposed 5 different kernels. We first investigated structural significances depending on substructure types with our previous walk kernel. Then, for accurate assessments, each kernel's performance was evaluated with the clean dataset, LLL. As a result, the walk-weighted subsequence kernel, which yielded the best performance, was selected for further experiments. We performed the walk-weighted subsequence kernel over the converted LLL data to figure out how the use of automatic parsed data affects the performance of the kernel in comparison with the clean dataset. Finally, the walk-weighted subsequence kernel was evaluated over the 5 converted PPI corpora.

Table 3 shows that the walk kernel operating with only *e*-walk information achieved a promising result on the 73.8 F-score, which indicates that *e*-walk information works better than *v*-walk in relation learning. This structural property was incorporated as prior knowledge in the extended dependency kernel. In addition, the kernel was modified to consider more extensive structural comparisons by allowing partial dependency path matches and counting the matches differently according to their types. As a result, the previous dependency kernel was significantly improved from 60.7 to 69.4 in F-score. However, there was still low since the kernel counts only the matches that preserve direct dependencies. Thus, we newly introduced string kernels, which could handle non-contiguous dependencies. From the spectrum kernel to the walk-weighted subsequence kernel, substructure types to be considered are incrementally augmented and the kernels gradually perform more comprehensive comparison of substructures enclosed by two shortest dependency strings.

**Table 3:** Performances of our kernels (LLL)

Kernel	Performance		
	PRE	REC	F-M
**walk kernel **[13]	72.5	83.3	77.5
**dependency kernel **[13]	58.6	62.9	60.7
**v-walk kernel**	51.4	66.6	58.0
**e-walk kernel**	71.9	75.9	73.8
**extended dependency kernel**	62.6	77.7	69.4
**spectrum kernel**	64.6	77.7	70.5
**fixed-length subsequence kernel**	72.5	83.3	77.5
**gap-weighted subsequence kernel**	68.4	72.2	70.2
**walk-weighted subsequence kernel**	**79.3**	**85.1**	**82.1**

The performances of kernels we suggested are shown. All kernels were trained and tested over clean data set (LLL training and test) and accessed by LLL external evaluation server.

As indicated in Table 3, even the spectrum kernel, which is the simplest string kernel, showed a better result than the dependency kernel. However, the fixed-length subsequence kernel showed a good performance, but the gap-weighted subsequence kernel, whose subsequence was penalized by its spread extent on strings, was unsatisfactory. The discounting by distance gap was not effective. Similarly, gap weighting for non-contiguous subsequences was not good as with the walk-weighted subsequence kernel. The best result was obtained with the walk-weighted subsequence kernel where their substructures were differently weighted according to their significance levels for learning. In the kernel, we classified string types into contiguous *e*-walk, contiguous *v*-walk, and non-contiguous subsequences and assigned different weights to each of them. The system performance was improved by 5% with the walk-weighted subsequence kernel over the original LLL data, as compared to the previous walk-based kernel (Table 3). According to the results by the LLL evaluation server, action-type interactions and negative interactions were recognized better in the walk-weighed subsequence kernel than in the previous walk-based kernel. We finally chose the walk-weighted subsequence kernel for further experiments.

Next, in order to compare the above result with ones on the data set made in an automatic way, we performed the same experiment over the converted LLL data using the walk-weighted subsequence kernel. For this, the kernel was trained on automatic-parsed LLL training data and then tested on automatic-parsed LLL test data. Table 4 shows the results evaluated by the LLL scoring server. Those were evaluated in terms of directed interactions. Consequently, the performance dropped to 68.5, which represents a 13.6% decrease in F-score, as compared to the use of perfectly parsed data.

**Table 4:** Results of our kernel on LLL and the converted LLL by [18]

Corpus	Precision	Recall	F-M
**Converted LLL**	68.5	68.5	**68.5**
**LLL**	79.3	85.1	**82.1**

Our walk-weighted subsequence kernel was trained on automatic-parsed LLL training data and then tested on automatic-parsed LLL test data. The result was evaluated by the LLL scoring server. It was compared with the result over clean LLL training/test set.

The walk-weighted subsequence kernel was also evaluated over the other 4 converted PPI corpora as shown in Table 5. It achieved an average F-score of about 67.46. In particular, the extraction performances over AIMed and BioInfer were relatively low compared to other corpora. Since, the two corpora include nested entities (Table 2) and their distributions of negative and positive examples are very unbalanced (Table 1), the performances were comparatively low although the two are large sized corpora in contrast to other corpora.

**Table 5:** Results on the 5 converted PPI corpora by [18]

Corpus	Positive Examples	negative	All examples	Precision	Recall	F-M
**AIMed**	1,000	4,834	5,834	61.42	53.26	56.59
**BioInfer**	2,479	7,174	9,653	61.84	54.24	57.58
**HPRD50**	163	270	433	66.67	69.23	67.82
**IEPA**	335	482	817	73.73	71.81	72.88
**LLL**	164	166	330	76.90	91.15	82.44

Our walk-weighted subsequence kernel was evaluated on 5 PPI corpora. We used the dependency parsed corpora by [18] for dependency information.

Consequently, the proposed kernel showed a bit low recall as compared to its precision on the automatic parsed datasets. In particular, recall rates were much lower over the AIMed and BioInfer. One of the main causes for low recalls might be inaccurate dependency representations on the converted corpora. As mentioned before, there were various difficulties including the converted data in experiments. Although we performed a rough disambiguation pre-processing to remove (1) multiple dependency categories, (2) unnecessary syntactic relations according to grouping named entity words, and (3) cycle relations from the converted corpora, some dependency relations were still ambiguous, which is a critical factor for training. The difficulties are summarized in Table 2. Thus, the performance can be enhanced by using other syntactic parser which is well adapted for this domain.

Although performance comparisons among systems would be more reliable than the LLL dataset since the 4 benchmark PPI corpora are much larger to show an advance and robustness of an approach, further discussions on the evaluation set and experimental settings will be necessary. Without benchmark test dataset and external evaluation, it is difficult to directly compare performances between approaches since there are substantial differences according to the data set used for training and testing, whether to include self interactions, preprocessing, or the data splitting strategy used in cross-validation. According to [11], the F-score on AIMed could increase up to 18% with a random splitting from a pool of all generated NE pairs.

### Comparisons with other systems

Table 6 shows comparisons with other systems that were trained and tested on the perfectly parsed LLL dataset. The systems were all accessed by the LLL external evaluation server. In the experiment, our system outperformed other systems. [28] applied sequence alignment and finite state automata to generate syntactic patterns for identifying genic interactions. [29] proposed the Markov Logic model to create a set of weighted clauses on the discourse representation structure which can classify pairs of interactive NEs. [6] created candidate relations from dependency parse trees by applying a small number of rules.

**Table 6:** Performance comparison with other systems (LLL)

PPI system	Method	Precision	Recall	F-M
[6]	rule based	68	78	72
[8]	sentence alignment & FST	50.0	53.8	51.8
[13]	walk kernel	72.5	83.3	77.5
[29]	Markov logic model	65.0	72.2	68.4
**Our system**	walk weighted subsequence kernel	**79.3**	**85.1**	**82.1**

The comparisons with other systems, which were accessed by LLL task's external evaluation, are shown.

Table 7 shows performance comparisons over AIMed with other systems. In the table, all systems except [9] used syntactic information by automated parsers. It is interesting that [9] achieved very competitive results in precision merely based on neighboring words context of entities. They did not exploit any structural information. On the other hand, the work of [11] using both syntactic and word context information showed a bit low recall as compared to its precision. [14] applied syntactic information to a graph kernel by considering dependencies outside of the path as well as dependencies on the shortest path between two entities. However, the precision was relatively lower than those obtained by other systems. [16] presented a composite kernel of previously suggested kernels. They combined the all-paths- dependency kernel by [14], the bag-of-words kernel by [11], and the subtree kernel by [20]. The system employed tag fixing and sentence splitting as preprocessing, and used the outputs of two parsers, Sagae and Tsujji's dependency parser and Miyao and Tsujii's Enju parser, which were retrained using the biomedical GENIA Treebank corpus. The system showed the current state-of-the-art results over other corpora as well as AIMed. Surprisingly, the recalls of the system were very high even in tricky corpora such as AIMed and BioInfer.

**Table 7:** Performance comparison with other systems (AIMed)

PPI system	Positive examples	All examples	Precision	Recall	F-M
[9]	1,000		**65.0**	46.4	54.2
[11]	1,068	5,631	64.3	44.1	52.0
[14]	1,000	5,834	52.9	61.8	56.4
[16]	1,000	5,834	55.0	**68.8**	**60.8**
**Our system**	1,000	5,834	61.4	53.3	56.6

The performances on AIMed of recent approaches were compared.

Table 8 shows comparison with other systems over the 5 PPI corpora. [18] presented the results over the converted 5 corpora using "RelEx" [6], a full parsing-based relation extraction system [18]. Their evaluation environment was not specifically described in the paper but they conducted the "RelEx" system on the same parsed corpora as that used in our system. Except HPRD50 domain, our kernel worked better than the system under the same syntactic information. The LLL extraction task shown in Table 8 is simpler than one in the original LLL challenge. They all performed 10-fold cross-validation on the LLL training set for evaluation and performances were assessed in terms of undirected interaction extraction.

**Table 8:** Performance comparison with other systems (5 PPI corpora)

PPI system	AIMed			BioInfer			HPRD50			IEPA			LLL		
	
	P	R	F	P	R	F	P	R	F	P	R	F	P	R	F
[14]	52.9	61.8	56.4	47.7	59.9	52.9	63.4	65.8	63.4	69.6	**82.7**	**75.1**	72.5	87.2	76.8
[16]	55.0	**68.8**	**60.8**	**65.7**	**71.1**	**68.1**	68.5	**76.1**	**70.9**	67.5	78.6	71.7	77.6	86.0	80.1
[18]	40	50	44	39	45	41	**76**	64	69	**74**	61	67	82	72	77
**Our system**	**61.4**	53.3	56.6	61.8	54.2	57.6	66.7	69.2	67.8	73.8	71.8	72.9	76.9	**91.2**	**82.4**

Our walk-weighted subsequence kernel was compared with other recent systems which were evaluated on 5 PPI corpora.

Table 9 shows averages of recalls, precisions and F-scores of the systems shown in Table 8 with respect to all the 5 PPI corpora. Notable is that the average precision of our system was higher than that in the other PPI extraction systems with a similar setting (i.e. the best F-value).

**Table 9:** Comparison of average performances

PPI system	All 5 PPI corpora		
	
	P	R	F
[14]	61.22	71.48	64.92
[16]	66.86	**76.12**	**70.32**
[18]	62.2	58.4	59.6
**Our system**	**68.12**	67.94	67.46

The average recalls, precisions and F-scores of the systems shown in Table 8 were computed with respect to all the 5 PPI corpora.

In conclusion, with respect to both the clean and converted LLL, we found increases in performance as compared to other systems. On the other hand, for the rest of fully automatically parsed corpora, we could not show significant improvements in F-score over other systems. As in many other researches, the extraction performances on AIMed and BioInfer, were worse than those on the other 3 corpora. In particular, recall rate requires further research to complement imperfect syntactic analyses derived from automated systems in the real world and insufficient information of the shortest path representation.

However, precisions of the proposed kernel were quite competitive in both clean dataset, and automated third-party datasets. As shown in the experiments, our approach worked better than other system under the same syntactic information environment or when accurate syntactic features were provided. Thus, the performance of the kernel is expected to be enhanced by the syntactic parser adapted for this task.

### Error analyses

**Figure 5:**
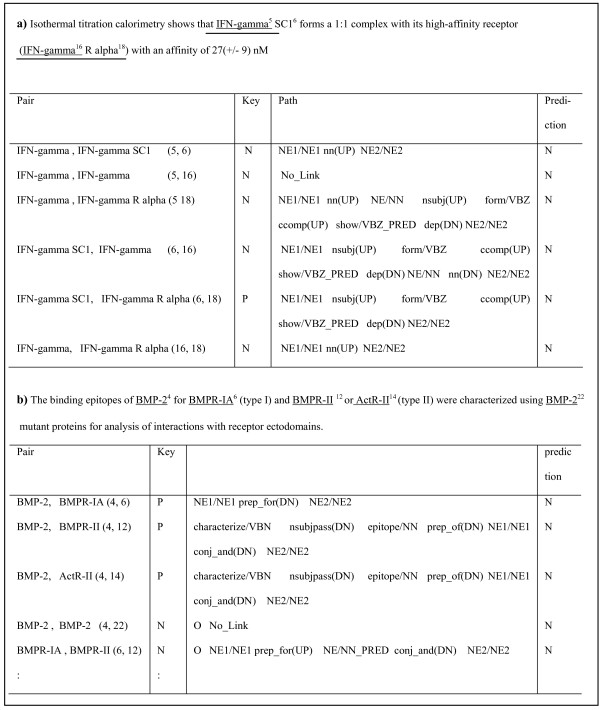
**Types of False Negative Examples**. Some false negative interactions to be recognized are shown. The NE pairs relating a) nested entities and b) single dependency shortest paths are the examples.

**Figure 6:**
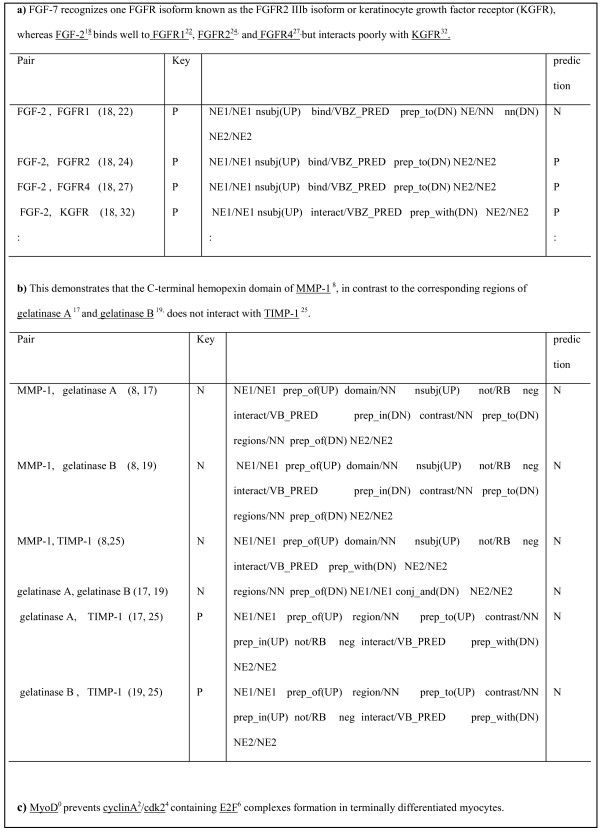
**Types of False Negative Examples (cont.)**. a) parsing errors, b) negations, and c) the cases which need information other than a sentence cause false negative interactions.

**Figure 7:**
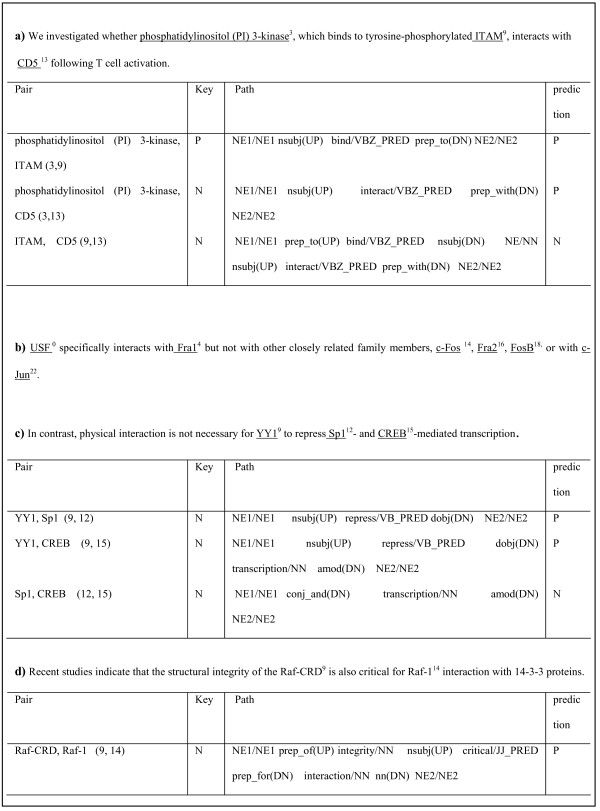
**Types of False Positive Examples**. Some false positive interactions to be filtered out are shown. The error types can be classified into a) and d) need more context, b) negation, and c) related words are not found on the shortest path.

In this section, we discuss the types of interactions that remain to be recognized and filtered out and how to improve the performance of the proposed walk-weighted subsequence kernel by analyzing errors. For convenience, we assumed the interactions to be undirected. In Figure 5, 6, and 7, "*N*" (negative) indicates that the pair has no relation and "*P*" (positive) accounts for a pair that has a relation. Figure 5 and 6 show some false negative interactions to be recognized.

First, many interactions regarding nested entities were filtered out. In fact, nested named entities are commonly encountered in biomedical text. For example, they account for 16.7% of all named entities of GENIA corpus. Currently, we allow all entities on nested entities. For instance, "*IFN-gamma*" and "*IFN-gamma SCI*" in Figure 5a are both considered. However, only one of them generally participates in an actual interaction. As mentioned earlier, since nested entities have the same structural and contextual information but prediction results need to be different, they prevent a proper learning. This can be one reason for the low performance. Thus, we need to restrict either the longest NE or the shortest NE for performance improvement.

Second, interactions related to single dependency shortest paths were filtered out. The single dependency shortest path implies one that is composed of two entities and a direct dependency between them, such as "*NE_conj_and_NE*" or "*NE_conj_or_NE*". In this case, we need further information other than the paths to retrieve correct interactions. To ensure sufficient information for relation extraction, we expanded the single dependency shortest paths. For instance, the shortest path between "*BMP-2*" and "*BMPR-1A*" in Figure 5b represented as "*NE/NE_prep_for(DN)_NE/NE*" requires contextual information outside the path such as "*binding*" to identify their interaction. In particular, the interactions of coordinated entities were often undetected because they commonly involve parsing errors. For the pairs of coordinated entities such as "*BMP-2*" and "*BMPR-II*" (4, 12) and "*BMP-2*" and "*ActR-II*" (4, 14) in Figure 5b, the distant useful words such as "*binding*" were still excluded in the shortest path, although the paths were extended to consider the surrounding contexts. After all, a more elaborate strategy for path extension with respects to single dependency path and coordination handling is required as future work. The path links that have no predicate should be reconsidered.

Third, some interactions are undetected due to parsing errors. In the biomedical domain, complex sentences containing coordination, apposition, acronym, clause dependency, or long dependency structures are very common. For instance, the first pair, "*FGF-2*" and "*FGFR1*" (18, 22) in the sentence of Figure 6a was not found due to incorrect dependency analysis in the form of "*nn(FGFR4-32, FGFR1-27)*". It implies that other supplementary information is required in addition to the shortest path representation to compensate for parsing errors often caused by the complicated coordination, apposition structures in biomedical texts. One way to reduce these can be efficient sentence splitting.

Our system still failed to identify some interactive pairs that occur with negation expressions. In Figure 6b, it correctly handled the negative pairs such as "*MMP-1*" and "*TIMP-1*" by negation processing but missed out interaction pairs such as "*gelatinase A*" and "*TIMP-1*" (17, 25), "*gelatinase B*" and "*TIMP-1*" (19, 25). Further studies should be performed in this negation processing. Finally, some cases need information other than a sentence, as shown in Figure 6c. In the sentence, the system cannot recognize the pair of "*cyclinA*" and "*cdk2*" (2, 4).

Figure 7 shows some types of false positive interactions that remain to be filtered out. In Figure 7a, the pair of "*phosphatidylinositol (PI) 3-kinase*" and "*CD5*" actually has no relation but our system recognized it as an interaction pair because of the substructure of "*interacts/VBZ_PRED prep_with(DN)*". It needs a much broader context including "*we investigated whether*~" for the correct extraction. Figure 7b shows a pair exemplifying a negation expression. In the sentence, the system detected all pairs as interactive pairs, but some pairs should have been filtered out. Our negation processing method could not cover the context of "*but not with*".

Figure 7c shows the interactions that are not detected with information on the shortest path alone. The shortest path in the sentence did not represent the context of "*is not necessary*". That is, words representing the interactions did not exist on the shortest path. Likewise, the interaction in Figure 7d should be filtered out. It requires a broad context such as "*interaction with 14-3-3 proteins*". It is one of the difficult cases to be filtered out.

### Discussion and future work

A more detailed investigation of other dependency parsers applied in PPI studies [11,16,19] should be performed in our future research. Although, according to the work of [19], 1% absolute improvement in parsing leads to 0.25% improvement in PPI extraction accuracy, it is quite important to obtain reliable syntactic information in the systems that fully depend on syntactic information without considering the bag-of-words context information. The critical point in biomedical text parsing is how well a parser handles coordination, apposition, and relative clauses which often cause erroneous results in PPI learning. In addition to a further improvement in parsing accuracy, the strategy for the shortest path extension should be improved to supplement incorrect syntactic analyses. Likewise, the method for the pairs, including nested entities and negation expressions, should be enhanced.

So far, we restricted the research to focus on structural kernels by using dependency information on the shortest path. However, the combination with the bag-of-words kernel can be a backup to compensate for the imperfect syntactic analyses derived from automated systems in the real world and insufficient information of the shortest path representation by including the neighboring words. The bag-of-context words kernel can improve recall rates. Additionally, studies on string kernels are possible because there can be a wide variety of string kernels depending on how the subsequences are defined.

## Conclusions

We presented kernel methods defined on the shortest dependency path for genic relation extraction. The dependency path between two NEs, which consists of the connections between words, is highly lexicalized. In this study, we started off with four drawbacks of our previous work in terms of e-walks, partial path matches, different significance levels of structures and non-contiguous paths and presented the revisions for the dependency kernel, variants of string kernels, and the walk-weighted subsequence kernel to effectively handle the drawbacks. The proposed kernels were experimented on the LLL shared task data and 5 PPI corpora. We achieved good performances only with substructures represented on the shortest path between entities.

## Methods

To handle the problems of the prior structural kernel, we first examined the effectiveness of each main feature for the walk kernel which showed the best performance in our previous work, and then modify the dependency kernel so that it can accept the features of the walk kernel and partial path matches.

In the modified version, we treat each type of substructures with different importance.

For this, we classify the types of substructures into several categories and enhance the learning performance by allowing different weights or counts according to the types of common dependency substructures that two relation instances share. Next, we treat the shortest path strings as strings and introduce some string kernels such as the spectrum kernel, subsequence kernel and gap weighted kernel. Finally, we suggest the walk weighted subsequence kernel, which can model not only the previous problems, but also non-contiguous structures and structural importance not covered by the previous kernels.

### Walk types

We start the kernel modification with the re-consideration of *walks *properties. In the walk kernel, the structural information is encoded with *walks *of graphs. Given *v *∈ *V *and *e *∈ *E*, a walk can be defined as an alternating sequence of vertices and edges, *v*_*i*_, *e*_*i, i+1*_, *v*_*i+1*_, *e*_*i+1, i+2*_, ..., *v*_*i+n-1*_. It begins with a vertex and ends with a vertex, where *V *and *E *are a set of vertices (nodes) and edges (relations), respectively. We took into consideration walks of length 3, v_*i*_, e_*i, i+1*_, v_*i+1*_, among all possible subsets of walks on the shortest path between a pair of NEs. We called it *v*-walk. Likewise, we defined *e*-walk which starts and ends with an edge, e_*i, i+1*_, v_*i+1*_, e_*i+1, i+2*_. It is actually not a walk defined in the graph theory, but we take *e*-walk to capture contextual syntactic structures as well. We utilized both lexical walks and syntactic walks for each of the *v*-walks and the *e*-walks. The lexical walk consists of lexical words and their dependency relations on a lexical dependency path like Figure 2c, and the syntactic walk, of POS and their dependency relations, on a syntactic dependency path, respectively. With this walk information, we can capture structural context information. This path-based walk representation is easy to incorporate structural information to the learning scheme because a path reflects the dependency relation map between words on it.

### Different properties of two walks

In this work, we focus on different structural properties of *v*-walk and *e*-walk. The *v*-walk shows a labeled relationship from a head to its modifier. Thus, it is related to a direct dependency relationship between two words or POS. On the other hand, *e*-walk describes the immediate dependency structure around a node. If a node is a predicate, then it has a close connection with the sub-categorization information which is important in semantic role labeling task for discovering the predicate-argument structure for a given sentence.

In Figure 2c, the *e*-walk of "*sub(UP)-control-comp_by (DN)*" shows the argument structure of the predicate verb, "*control*". In this case, one entity fills the "*subject*" argument of "*control*" and the other entity directly or indirectly fills the "*comp_by*" role. If an instance holds such dependency structure with respect to the predicate of "*control*", it is very likely that two NEs in the structure have a genic relation. The semantic relations among predicates and their modifiers are clearly helpful for relation extraction. According to [28], the F-score was improved by 15% when incorporating semantic role information into the information extraction system.

Thus, we evaluated each walk type's contribution to the interaction extraction. For this, we conducted the experiment by restricting the walk kernel to operate with a single walk type. As shown in Table 3, we could achieve a quite competing result only with *e*-walk information. Clearly, this result demonstrates that *e*-walk contributes more to the overall similarity for relation learning than *v*-walk since it is related to semantic role information. However, the *e*-walk style structural information is excluded in the previous dependency kernel, which is one of the reasons for the low performance. Therefore, such information should be considered as prior knowledge, and be regarded as more significant structures, among the subpaths.

### Modified dependency kernel

The dependency kernel directly computed the structural similarity between two graphs by counting common subgraphs. However, our previous dependency kernel rigorously focused on *v-walk*, so the direct dependencies between pairs of nodes and *e-walk *style structural information was excluded. Two nodes match when the two nodes were the same and their direct child nodes and the dependency types from the nodes to their direct child nodes matched. Thus, we extend the kernel by allowing the possibility of partial matches besides *e*-walk with an extra factor ensuring that the partial matches have lower weights than complete path matches.

In the extended dependency kernel, partial matches such as single word/POS matches and node-edge or edge-node matches are counted, as well as *v*-walks. Moreover, the matches are all differently weighted. Before we explain the matching function, we will introduce some notations. For each node *x*, *word*(*x*) is the word at a certain node and POS(*x*) is the POS of the node. *children*_*w*_(*n*) denotes word dependency list of word *n *and *children*_*p*_(*p*) refers to POS dependency list of POS *p. children*_*w*_(*n*) is the set of (*relation*, *word*) pairs which are direct modifiers of *n*. In a similar way, *children*_*p*_(*p*) is the set of (*relation*, *pos*) pairs which are direct modifiers of POS *p*. In addition, *sc*_*w*_(*n*_*1*_, *n*_*2*_) and *sc*_*p*_(*p*_1_, *p*_2_) denote the set of common dependencies between two subgraphs rooted at *n*_*1 *_and *n*_*2*_, and POS *p*_1 _and *p*_2_, respectively. We can define the sets of common dependencies between two graphs as follows:(3)

That is, (*x*, *y*) can be an element of the set of *sc*_*w*_(*n*_*1*_, *n*_*2*_) only when the direct child nodes of two parent nodes, *x *and *y*, are the same word and have the same dependent relation with their parents *n*_*1 *_and *n*_*2 *_as well. For subcategorization information, *subcat*_*w*_(*x*) is used to refer to the sub-categorization pair of a word *x *which is composed of the left and right edge of it. That is the same information with *e*-walk. The matching function *C*_*w*_(*n*_*1*_, *n*_*2*_) is the number of common subgraphs rooted at *n*_*1 *_and *n*_*2*_.

The similarity is recursively computed over their common dependency child word pairs in the set *sc*_*w*_(*n*_*1*_, *n*_*2*_), starting from root nodes. As a result, we can calculate *C*_*w *_as follows:(4)

In order to count common subgraphs with considering their structural importance, the matching function was devised. In the definition, if the set of common dependency child word pairs is empty but the two nodes have the same sub-categorization value, then the matching function returns 3.0. If there is no child of *n*_1 _or *n*_2 _but two nodes are the same words, then *C*_*w*_(*n*_*1*_, *n*_*2*_) returns 1.0. In case that there is no child of *n*_1 _or *n*_2 _and two nodes are different words, *C*_*w*_(*n*_*1*_, *n*_*2*_) returns 0. The last two definitions recursively call *C*_*w *_with respect to their common dependency word pairs in the set *sc*_*w*_(*n*_*1*_, *n*_*2*_) but *C*_*w *_is weighted with a larger value if the two nodes share the same subcategorization information.

Such isomorphism between two graphs is identified in terms of common POS dependencies in addition to common word dependencies. In the similar way, *C*_*p*_(*p*_*1*_, *p*_*2*_) is applied for common POS dependency subgraphs rooted at POS *p*_*1 *_and *p*_*2*_. However, in case of syntactic dependency path, the subcategorization information is excluded as follows:(5)

Since *C*_*w *_and *C*_*p *_have different properties that *C*_*w *_is related to lexical and *C*_*p*_, to morpho-syntactic subgraphs, *C*_*w *_is more weighted than *C*_*p*_. Finally, the dependency kernel evaluates the similarity of two graphs by the composition of syntactic dependencies and lexical dependencies as follows:(6)

The formula (6) enumerates all matching nodes of two graphs, *d*_*1 *_and *d*_*2*_. It is a summation of common word dependency subgraphs and common POS dependency subgraphs between two graphs.

As a result, the F-score was improved from 60.4 to 69.4 on LLL dataset (Table 3), compared with the previous dependency kernel. The uses of partial path match and subcategorization information were helpful but the result is still worse than that by the walk kernel. In order to maintain direct dependency structures, this kernel excluded the non-contiguous sub-paths on the shortest path which can be important in the relation learning. Thus, we introduce string kernels to handle such non-contiguous subpaths.

### String kernels

In this section, we will look at the string kernels from various structural perspectives. First of all, we will briefly introduce concepts and notations for string kernels. The string kernel was first addressed in the text classification task by [30]. The basic idea is to compare text documents by means of substrings they contain: the more substrings in common, the more similar they are. A string is defined as any finite sequence of symbols drawn from a finite alphabet and string kernels concern occurrences of subsequences or substrings in strings. In general, for a given string s_*1 *_s_*2*_...s_*n*_, a *substring *denotes a string, s_*i*_s_*i+1*_...s_*j-1*_s_*j*_, that occurs contiguously within the string, while a *subsequence *indicates an arbitrary string, s_*i*_s_*j*_...s_*k *_whose characters occur contiguously or non-contiguous.

So far, we re-represented the shortest path strings with meaningful substructures such as walks. In this work, we also project the shortest path string like Figure 2c to a string itself and directly compare the strings. On the basis of our data representation, nodes and edges of a shortest path string correspond to alphabets of a string. That is, a finite alphabet set, consists of word or POS and dependency relation symbols of shortest path strings and string kernels operate on the shortest path strings. The kernels consider both lexical shortest path string and syntactic shortest path string. We gradually enlarge the kernels to perform a more comprehensive comparison between the two shortest path strings, from the spectrum kernel to the weighted subsequence kernel.

### Spectrum kernel

First, we performed string comparisons with a simple string kernel. One of the ways to compare two strings is to count how many *p*-length contiguous substrings they have in common. It is called the spectrum kernel of order *p *or *p*-spectrum kernel. We borrowed the notation from [27]. Such *bag- of-characters *representation is the most widely used in natural language processing. However, the major shortcoming is the structural simplicity that all features represent only local information. In Equation (7), *K*_*s*_(*s*_1_, *s*_2_) denotes the number of common *p*-substrings between two shortest path strings, *s*_1 _and *s*_2_.(7)

The string *s*(*i*: *i + p *) means the *p*-length substring *s*_*i*_...*s*_*i + p *_of *s*. In this work, we fixed the order of spectrum as 3 and summed *K*_*s *_of lexical dependency path string and *K*_*s *_of syntactic dependency path string for the common substring counting. With this kernel, we can consider the substructure as shown in Figure 1f. As a result, we achieved the F-score of 70.5 on LLL data (Table 3). In spite of its structural simplicity, the result was quite promising. It was better than the performance of the extended dependency kernel. We could obtain a reasonable performance only with contiguous dependencies on the shortest path string.

### Fixed-length subsequence kernel

In the spectrum kernel, substructures such as "*stimulate_obj(DN)~comp_from(DN)*", which has gaps between them, is excluded in the structural comparison. In order to cover the substructures, we tested the subsequence kernel that the feature mapping is defined by all contiguous or non-contiguous subsequences of a string. Unlike the spectrum kernel, the subsequence kernel allows gaps between characters. That is, some characters can intervene between two matching subsequences. Thus, this kernel can explain the substructures like Figure 1g. The substructure of "*stimulate-obj(DN)~comp_from(DN)*" can match phrases such as "*stimulate-obj(DN)-any other noun-comp_from(DN)*" which use other nouns instead of "*transcription*". The advantage of the kernel is that we can exploit long-range dependencies existing on strings. Likewise the spectrum kernel, we reduce the dimension of the feature space by only considering fixed-length subsequences. This kernel is defined via the feature map from the space of all finite sequences drawn from to the vector space indexed by the set of *p*-length subsequences derived from A. We will define A^*p *^as the set of all subsequences of length *p*. We denote the length of the string, *s = s*_1_*s*_2_...*s*_|*p*| _by |*p*|. Also, *u *indicates a subsequence of s if there exist an index sequence i = (i_1_...i_|u|_) with 1 ≤ *i*_1 _< ... <*i*_|*u*| _≤ |*p*| such that *u*_*j *_= *s*_*i *_for *j *= 1, ⋯, |*u*|. We use a boldface letter i to indicate an index sequence i_1_...i_|u| _for a string and the subsequence *u *of a string *s *is denoted by u = s[i] for short. That is, *u *is a subsequence of *s *in the position indexed by i and equals to .

Then, the feature coordination function *ϕ*_*u*_(*s*) is used to denote the count of how many times substring *u *occurs as a contiguous and non-substring in the input string *s. I*_*p *_is the index sequences set of length *p*. *ϕ*_*u*_^*p*^(*s*) indicates the count of how many times substring *u *of length *p *occurs. Consider two strings, *s *and *t *to be compared that have the same length *p*, where the feature space is generated by all subsequences of length *p *derived from shortest path strings to be classified. Then, the overall inner product between them can be expressed as follows:(8)

We choose 3 as the length parameter *p*. Despite the positive aspect of the subsequence that it considers non-contiguous subsequences as well as contiguous substrings, the performance was not satisfactory. It has improved over the spectrum kernel to some extent, but it was the same value with the walk kernel as F-score 77.5 on LLL which showed the best result in our previous study.

### Gap-weighted fixed length subsequence kernel

In the subsequence kernel, all substructures are equally counted. It does not matter whether the subsequence is continuous, non-continuous or how spread out the occurrences of subsequences are. Thus, the gap-weighted subsequence kernel is tested so as to reflect degree of the contiguity of the subsequence to the subsequence kernel. In this kernel, two substructures of "*stimulate-obj(DN)-transcription*" and "*stimulate~ comp_from(DN)-promoter*" have different weights. For the purpose of tuning the weight to reflect how many gaps between characters there are, a decay factor, *λ*(0 <*λ *≤ 1) is introduced. It can penalize non-contiguous substring matches. That is, the further apart the beginning and the end in a substring are, the more it is penalized. Contiguous substring matches are assumed to be coherent and affect more the overall meaning of shortest path string. The feature coordination function is changed into a weighted count of subsequence occurrences as follows:(9)

The count is down-weighted by the total length of gaps. *l*(**i**) denotes the span length of indices **i**, i_|u| _-i_1_+1. The similarity value between two subsequences are decreased by the factor of *l*(**i**) and *l*(**j**), reflecting how spread out the subsequences are. The inner product between two strings *s *and *t *over A^*p *^is a sum over all common fixed-length subsequences that are weighted according to their frequency of occurrence and lengths.(10)

That is, this kernel function computes all matched subsequences of *p *symbols between two strings and each occurrence is weighted according to their span. In general, a direct computation of all subsequences becomes inefficient even if we use a small value of *p*. For an efficient computation, the dynamic programming algorithm by [30] was used. In this paper, we will not explain the details about the efficient recursive kernel computation method. We set the lambda as 0.5 and the index set is fixed as *U *= A^3 ^(three node or edge phrases on the shortest path string). If we choose *λ *as 1, the weights of all occurrences will be 1 regardless of *l*. In that case, the kernel is equivalent to the fixed length subsequence kernel that identically counts all common subsequences as 1. As a result, the F-score (70.2) was lower than the subsequence kernel even though this kernel can offer a more comprehensive weighting scheme depending on the dependency distance of each subsequence. The inclusion of gap weighting to substrings was not much effective.

### Walk-weighted fixed length subsequence kernel

In order to improve the gap-weighted subsequence kernel, we devise the walk-weighted subsequence kernel which can handle structural properties differently in addition to the consideration of contiguous and non-contiguous substring. Like the gap-weighted subsequence kernel, this kernel assigns different weights for each subsequence. However, it assigns the weights of subsequences not by the lengths of gaps, but by their type. We set more weights to contiguous subsequences than to non-contiguous subsequences since they are coherent and can affect more the overall meaning of shortest path string. Also, *e*-walks get more weights than *v*-walks so that highlight semantic role information. Similarly, this kernel also considers subsequences of length 3.(11)

The formula (11) means that the kernel assigns 3.0 for common contiguous *e*-walk substrings, 2.0 for common contiguous *v*-walk substrings. For non-contiguous subsequences, they can be penalized by gap-weights, but the performance was the best when we set the lambda to 1.0. Thus, in our experiments, 1.0 was also allocated to non-contiguous subsequences regardless of their gap. The significance values can take into account the types of substructures and we experimentally set the significance values for the best F-value.

As a result, this kernel showed the best performance (F-score 82.1) for the extraction of genic relation on the LLL data. This result demonstrates that the use of carefully designed weighted string kernels in terms of types of common subsequences is very effective on learning of a structured representation.

## Authors' contributions

SHK and JTY carried out PPI studies and suggested overall idea of this manuscript, and the system design. They performed the experiments and analyzed the results. JHY and SP participated in the system design and helped to draft the manuscript. All authors read and approved the final manuscript.
